# Astaxanthin suppresses the metastasis of colon cancer by inhibiting the MYC-mediated downregulation of microRNA-29a-3p and microRNA-200a

**DOI:** 10.1038/s41598-019-45924-3

**Published:** 2019-07-01

**Authors:** Hye-Youn Kim, Young-Mi Kim, Suntaek Hong

**Affiliations:** 1 Laboratory of Cancer Cell Biology, Department of Biochemistry, Gachon University School of Medicine, Incheon, Republic of Korea; 2 Department of Health Sciences and Technology, GAIHST, Gachon University, Incheon, 21999 Republic of Korea

**Keywords:** Metastasis, Drug development

## Abstract

Colorectal cancer (CRC) is the third most common cancer, and is associated with a high percentage of cancer-related death globally. Furthermore, the success rate of therapeutic treatment for CRC patients mainly depends on the status of metastasis. Therefore, novel drugs or therapeutic techniques should be discovered for the treatment of metastatic CRC. In this study, we selected Astaxanthin (AXT), one of the most common carotenoids, as a novel metastasis inhibitor through high-throughput drug screening based on invadopodia staining, and confirmed the anti-migratory and anti-invasive activity of AXT. We demonstrated that AXT increases miR-29a-3p and miR-200a expression, and thereby suppresses the expression of MMP2 and ZEB1, respectively. As a result, AXT represses the epithelial-mesenchymal transition (EMT) of CRC cells. Through the mechanistic study, we identified that AXT shows anti-metastatic activity through the transcriptional repression of MYC transcription factor. Finally, we also confirmed that AXT suppresses the *in vivo* metastatic capacity of colon cancer cell using mouse model. Collectively, we uncovered the novel function of AXT in the inhibition of EMT and invadopodia formation, implicating the novel therapeutic potential for AXT in metastatic CRC patients.

## Introduction

Colorectal cancer (CRC) is the most frequently occurring human malignancy, its incidence in males and females being the third and second highest, respectively, in the world^1^. While the 5-year survival rate for early-stage CRC patients is about 90%, it is only 10 to 15% for metastatic CRC patients. Metastasis of colon cancer cells is the leading cause of cancer-related deaths in CRC patients. Therefore, targeting of metastasis is an important strategy to improve the therapeutic outcomes^2,3^. Metastasis is a very complex and multi-step process, and the progression of metastasis research has largely been expended on a variety of cancers^4^. However, there has been limited research on the molecular mechanisms for regulating the metastatic process of CRC. Metastasis comes when cancerous cells penetrate the extracellular matrix (ECM) to leave the primary tumor for intravasation into blood and lymphatic vessels^5^. During metastasis, invasive cancer cells degrade ECM by forming unique F-actin-rich protrusions called invadopodia^6,7^, which process was identified in a number of metastatic cancer cell lines, such as breast, prostate, melanoma, and fibrosarcoma^8^. Recent studies also reported the molecular crosstalk between invadopodia formation and metastasis in various tumors, and considered them a key step for treatment^9–11^.

Astaxanthin (3,3′-dihydroxy-β-carotene-4,4′-dione, AXT) is a xanthophyll carotenoid commonly found in plants and seafoods^12^. Generally, carotinoids consist of hydrocarbon (β-carotene, α-carotene, γ-carotene, and lycopene), and oxygenated (violaxanthin, neoxanthin, fucoxanthin, lutein, zeaxanthin, astaxanthin, and canthaxanthin) carotinoids have been shown to be important bioactive compounds. The United States Food and Drug Administration and the European Commission approve AXT as color additives for food dye. Among carotinoids, AXT shows an exclusive aspect, due to its wide variety of biologic effects against cancer, inflammation, and aging^13^. Recent studies have reported that AXT has anti-cancer activity in various type of cancers. AXT suppresses oral carcinomas by inducing apoptosis through the inhibition of Erk/MAPK and PI3K/Akt signaling^14^. AXT was also confirmed to suppress AOM/DSS-induced colon inflammation and carcinogenesis in animal model through inhibiting the NF-κB pathway^15^. Furthermore, AXT reduces the metastasis of cancer cells by decreasing the expression of MMPs, which are critical for the metastasis of cancer cells through degrading the ECM^16,17^. Although AXT has been reported to inhibit metastasis in a variety of cancers, the detail mechanisms underlying metastatic potential of CRC remain to be studied.

In this report, we identified AXT as a novel metastasis inhibitor through high-throughput drug screening, and validated the inhibitory effect of AXT on invadopodia formation in colon cancer cells. Furthermore, AXT increases the expression of microRNA-29a-3p (miR-29a-3p) and miR-200a by the transcriptional repression of MYC oncogenic transcriptional factor, thereby abrogating their downstream target genes, MMP2 and ZEB1, and consequently suppressing epithelial-mesenchymal transition (EMT) and metastasis. This study provides the supporting data that AXT inhibits the metastasis of colon cancer through the suppression of invadopodia formation. Collectively, our findings suggest that AXT could have therapeutic activity in metastatic CRC patients.

## Results

### Astaxanthin inhibits invadopodia formation and metastatic capability in colon cancer cells

**Figure 1:**
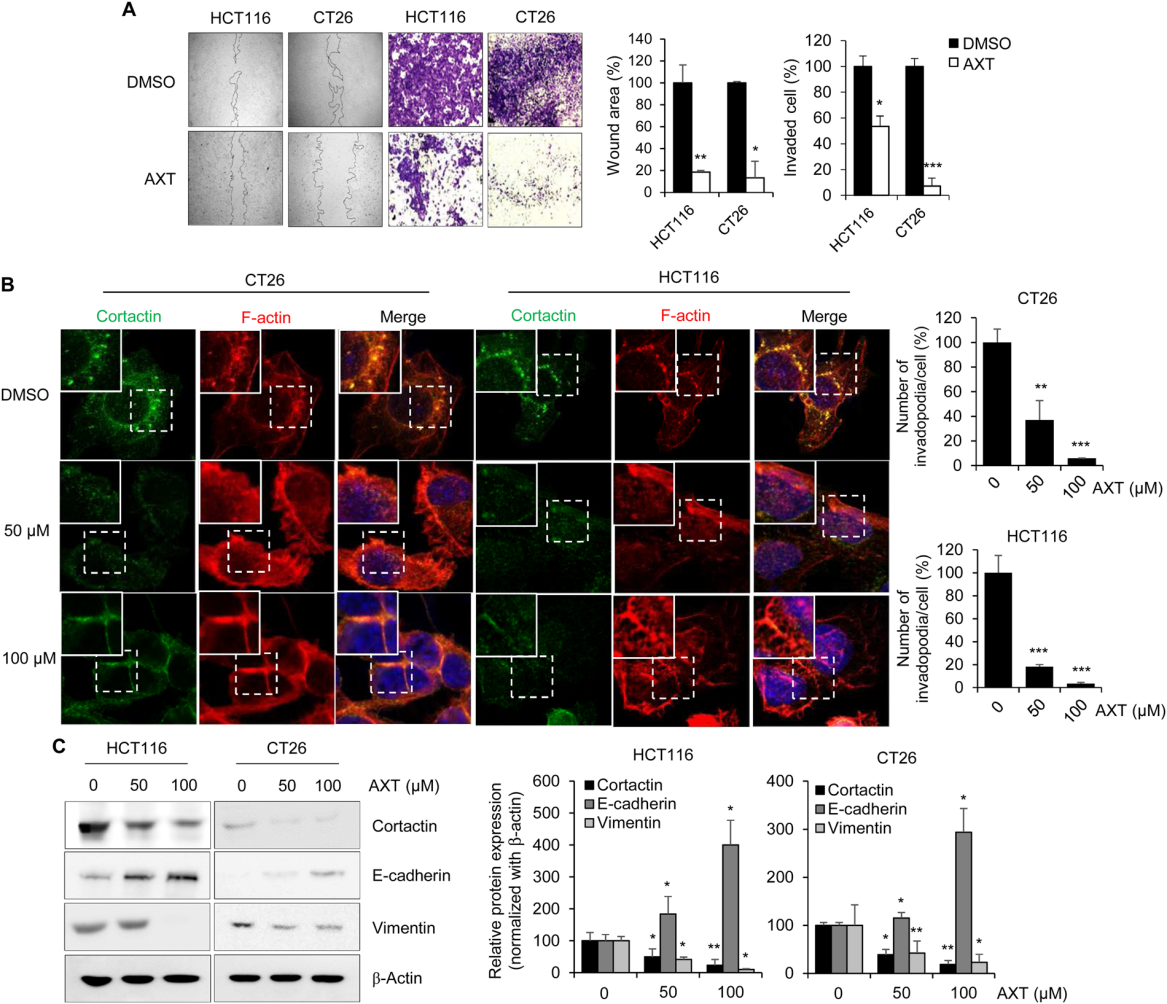
Astaxanthin inhibits the invadopodia formation and metastatic capacity in colon cancer cells. (**A**) To check the invasive activity of colon cancer cells, wound healing and trans-well matrigel assay were performed with AXT (50 µM) or DMSO-treated colon cancer cells. Images were captured with microscopy 24 h after treatment of AXT or DMSO. The migrated and invaded cells were quantified with Image J software to compare with control. (**B**) To evaluate the invadopodia formation, colon cancer cells were treated with AXT or DMSO with the indicated concentrations for 24 h. Cells were fixed and labeled for F-actin (red) and Cortactin (green) as invadopodia markers. Scale bar, 50 μm. Staining intensity was compared with Image J program from at least three fields. (**C**) Invadopodia (Cortactin) and EMT markers (E-cadherin and Vimentin) were detected in AXT-treated colon cancer cells with specific antibodies. The β-actin band was validated as normalization control. Expression level of specific protein was measured with densitometry, and presented as relative density. Values are mean ± SD from three independent experiments. *P < 0.05; **P < 0.01; ***P < 0.001.

To identify the novel metastasis inhibitor against CRC, we performed high-throughput drug screening based on invadopodia staining (Fig. S1 of the Supplementary Information (SI)). Among candidates, AXT shows the strongest activity on the reduction of invadopodia formation. To validate whether AXT could potentially suppress invadopodia formation and the consequent suppression of metastasis, metastatic colon cancer cells (CT26 and HCT116) were treated with AXT for 24 h, followed by metastatic analysis through wound and transwell matrigel invasion assays. As shown in Fig. 1A, the abilities of migration and invasion in AXT-treated cells were significantly inhibited, as compared with in DMSO-treated cells. To examine whether AXT inhibits invadopodia formation, CT26 and HCT116 cells were treated with AXT or DMSO, labeled for F-actin and Cortactin as invadopodia markers (Fig. 1B). Furthermore, the activity of matrix degradation by invadopodia was significantly decreased in AXT-treated CT26 and HCT116 cells (Fig. S2 of the SI). Based on MTT assay and *in vivo* xenograft model, AXT did not show metastasis-suppressing activity by growth inhibition (Fig. S3A–D of the SI).

Because the EMT process is an important step for cancer cells to metastasize^18^, we confirmed whether AXT modulates the EMT in colon cancer cells. Western blotting analysis suggested that the expressions of epithelial marker, E-cadherin were enhanced in AXT-treated HCT116 and CT26 cells (Fig. 1C). In contrast, the mesenchymal marker, Vimentin, was suppressed in AXT-treated cells in a dose-dependent manner. In addition, another invadopodia marker, Cortactin, was also decreased in AXT-treated cells (Fig. 1C). These results showed that AXT is a potential inhibitor of metastasis through the suppression of invadopodia formation and EMT.

### Astaxanthin suppresses MMP2 activity through upregulation of miR-29a-3p

**Figure 2:**
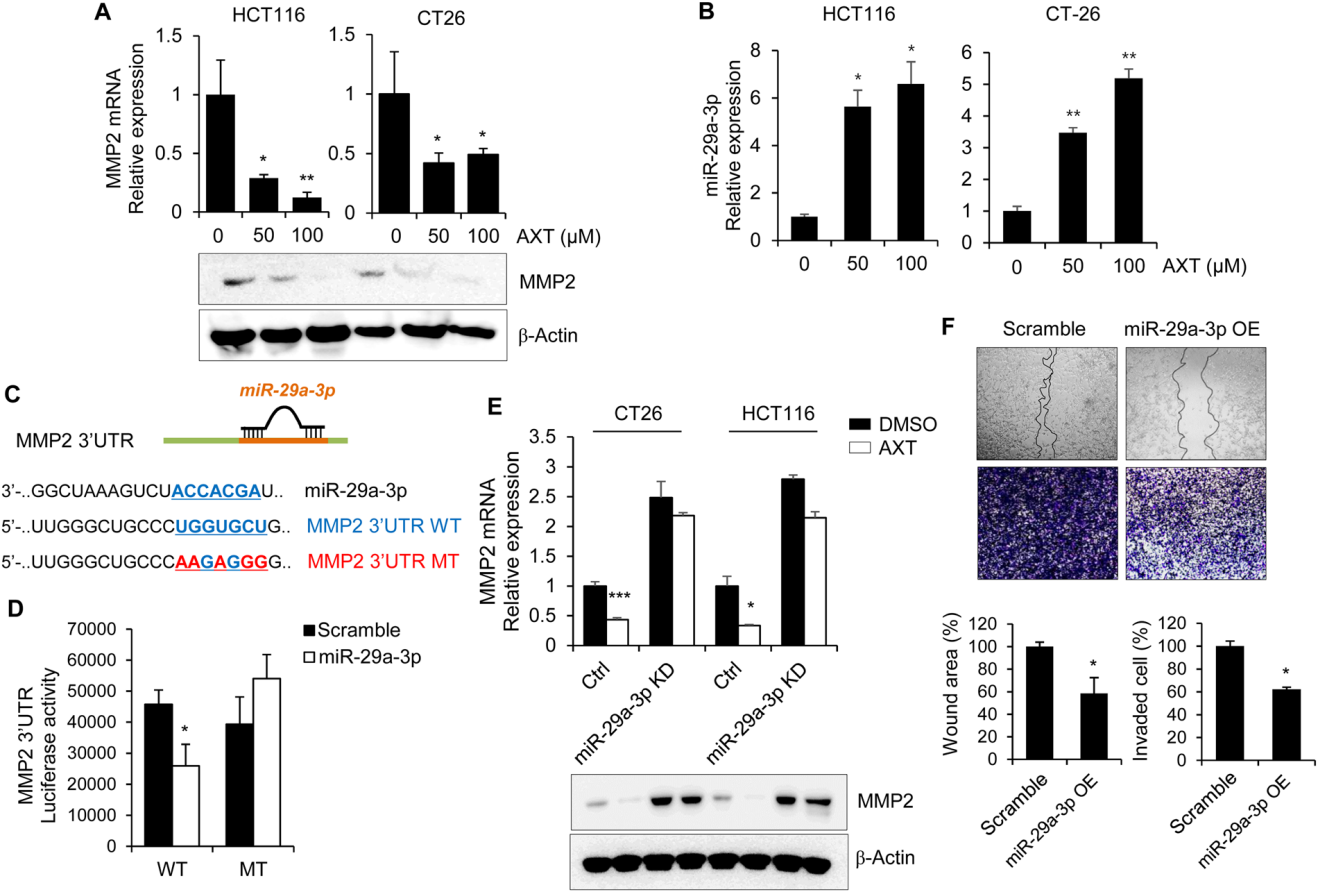
Astaxanthin suppresses the expression of MMP2 through upregulation of the miR-29a-3p. (**A**) To check the effect of AXT on MMP2 expression, cells were treated with AXT at the indicated doses. After isolating RNA and protein, expression of MMP2 was detected with qRT-PCR and western blot in AXT-treated colon cancer cells to compare with control cell. (**B**) To determine the expression level of miR-29a-3p in AXT-treated colon cancer cells, total RNA was purified, and examined with miRNA-specific qRT-PCR. Level of 18S RNA was detected as housekeeping control. (**C**) Schematic showing the miRNA binding sequence in the 3′UTR of MMP2 gene, which is targeted bymiR-29a-3p. The 3′UTR region of MMP2 was amplified, and cloned into the pMIR-REPORT luciferase reporter construct. Mutant having nucleotide changes at miR-29a-binding sites was generated as indicated. (**D**) Luciferase reporter activity was measured with pMIR-REPORT construct including wild type or mutant MMP2 3′UTR into miR-29a-3p-overexpressed CT26 cell. Incubated cells were collected to compare the relative luciferase activity. The raw data were normalized with β-galactosidase activity. (**E**) To confirm the effect of miR-29a-3p on MMP2 suppression by AXT, miR-29a-3p expression was suppressed by transfecting with miR-29a-3p sponge. The expression of MMP2 mRNA and protein was determined via qRT-PCR and western blot. The *Cyclophilin* gene and β-actin were used as loading control, respectively. (**F**) Wound assay and invasion assay were performed with miR-29a-overexpressing CT26 cells. The percentage of wound closure or invaded cells was compared with non-treated cell. *P < 0.05; **P < 0.01; ***P < 0.001.

Matrix metalloproteinases (MMPs) are enriched in invadopodia, and capable of degrading the ECM and basement membrane (BM)^18^. Numerous studies suggested that expression of MMPs can be used as prognostic markers in colorectal cancer^19,20^. To confirm the effect of AXT on MMPs expression, HCT116 and CT26 cells were treated with AXT of (50 and 100) µM for 24 h. As shown in Fig. 2A, both mRNA and the protein expression of MMP2 were suppressed in AXT-treated HCT116 and CT26 cells.

Previous studies have reported that miR-29 members suppress cancer angiogenesis, invasion, and metastasis in various tumors by direct targeting MMP2^21,22^. Therefore, we examined the effect of AXT on miR-29 family expression in colon cancer cells. The expression of miR-29a-3p is significantly upregulated in AXT-treated HCT116 and CT26 cells (Fig. 2B). To further validate whether MMP2 is a direct target of miR-29a-3p, luciferase assay constructs were generated with putative miR-29a-3p binding site in 3′UTR of MMP2 mRNA, including wild type and mutant form (Fig. 2C). As shown in Fig. 2D, miR-29a-3p inhibited the luciferase expression of MMP2 wild type 3′UTR form, but not the mutant one, indicating that miR-29a-3p reduces the MMP2 mRNA stability through direct targeting the 3′UTR of MMP2 mRNA.

To validate the activity of miR-29a-3p on the abrogation of MMP2 by AXT, we generated miR-29a-3p-knockdown colon cancer cells by transfecting miR-29a-3p sponge construct. Inhibition of miR-29a-3p activity caused no significant suppressive effect of AXT on MMP2 mRNA and protein expression compared to control (Fig. 2E). To determine the effect of miR-29a-3p on the metastatic capacity of colon cancer cells, we generated miR-29a-3p overexpressed CT26 cell (Fig. S4A of the SI). Restoration of miR-29a-3p significantly reduced the mRNA and protein level of MMP2 (Fig. S4B of the SI). Furthermore, modulation of miR-29a-3p also markedly reduced the migration and invasion capacities of CT26 cells (Fig. 2F). These results indicate that increased miR-29a-3p by AXT suppresses MMP2 expression by direct targeting 3′UTR of mRNA and influences the migration and invasion activities of colon cancer cells.

### Astaxanthin inhibits the expression of ZEB1 through enhancement of miR-200a

**Figure 3:**
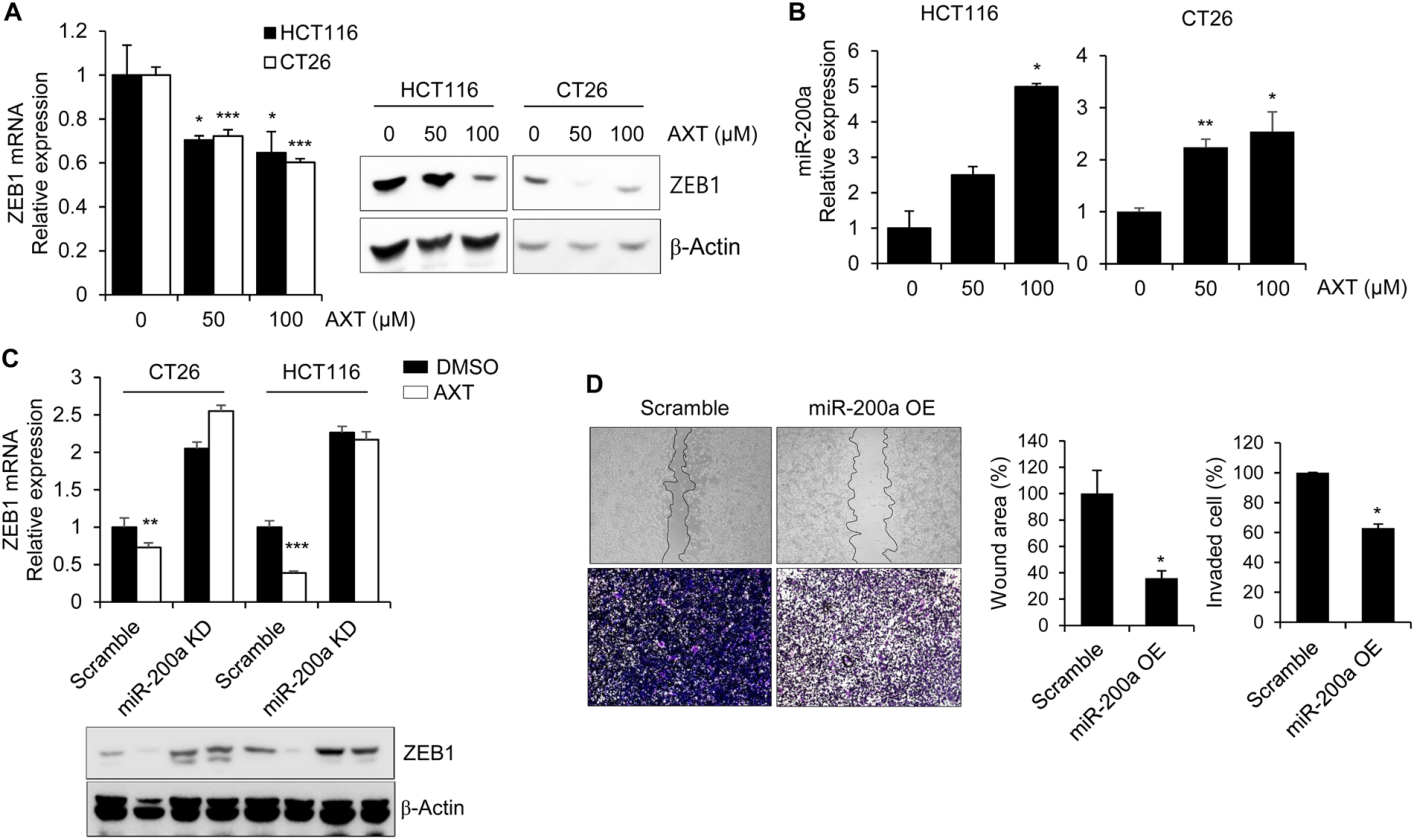
Astaxanthin inhibits the expression of ZEB1 through the enhancement of miR-200a. (**A**) To examine the effect of AXT on ZEB1 expression, cells were treated with AXT at the indicated doses. After isolating RNA and protein, expression of ZEB1 was detected with qRT-PCR and western blot and compared with control cell. (**B**) To determine the expression level of miR-200a in AXT-treated colon cancer cells, total RNA was isolated and examined with miRNA-specific qRT-PCR. Level of 18S RNA was detected for normalization. (**C**) To evaluate the inhibitory effect of miR-200a on ZEB1 by AXT, miR-200a expression was reduced by treating with miR-200a sponge. The expression level of *ZEB1* mRNA and protein was determined by qRT-PCR and western blot. The *Cyclophilin* gene and β-actin were used as loading control, respectively. (**D**) Wound closure and invasion assay were performed with miR-200a-overexpressing CT26 cell. The percentage of wound closure or invaded cells was compared with non-treated cell. *P < 0.05; **P < 0.01; ***P < 0.001.

ZEB1 is a critical EMT activator in many human cancers, and promotes the metastasis of cancer cells as E-cadherin repressor^23,24^. As shown in Fig. 3A, ZEB1 mRNA and protein expression were dramatically suppressed in AXT-treated colon cancer cells. Previous studies showed that ZEB1/ZEB2 and miR-200 family negatively regulate each other, which has important implication for EMT and tumor metastasis^25,26^. To identify the molecular links how AXT modulates ZEB1 expression, we examined whether AXT regulates miRNA-200 family. To confirm our hypothesis, miRNA-200 family expression was checked in AXT-treated colon cancer cells, and confirmed that only miR-200a expression was increased by AXT treatment (Fig. 3B).

To validate the effect of miR-200a on the abrogation of ZEB1 by AXT, we generated miR-200a knockdowned colon cancer cells by transfecting miR-200a sponge. Knockdown of miR-200a expression causes no significant suppressive effect of AXT on ZEB1 mRNA and protein expression compared to control (Fig. 3C). To determine the effect of miR-200a on the metastatic capacity of colon cancer cells, we generated miR-200a-overexpressed CT26 cell. Overexpression of miR-200a inhibited the mRNA and protein level of ZEB1 in colon cancer cells (Fig. S3 of the SI). In addition, the migration and invasion activity were significantly suppressed in miR-200a overexpressed CT26 cells compared with control cells (Fig. 3D). Taken together, these findings clearly indicate that AXT suppresses ZEB1 by the restoration of miR-200a level, and influences the invasive capability of colon cancer cells.

### Astaxanthin negatively regulates MYC oncogenic transcription factor

**Figure 4:**
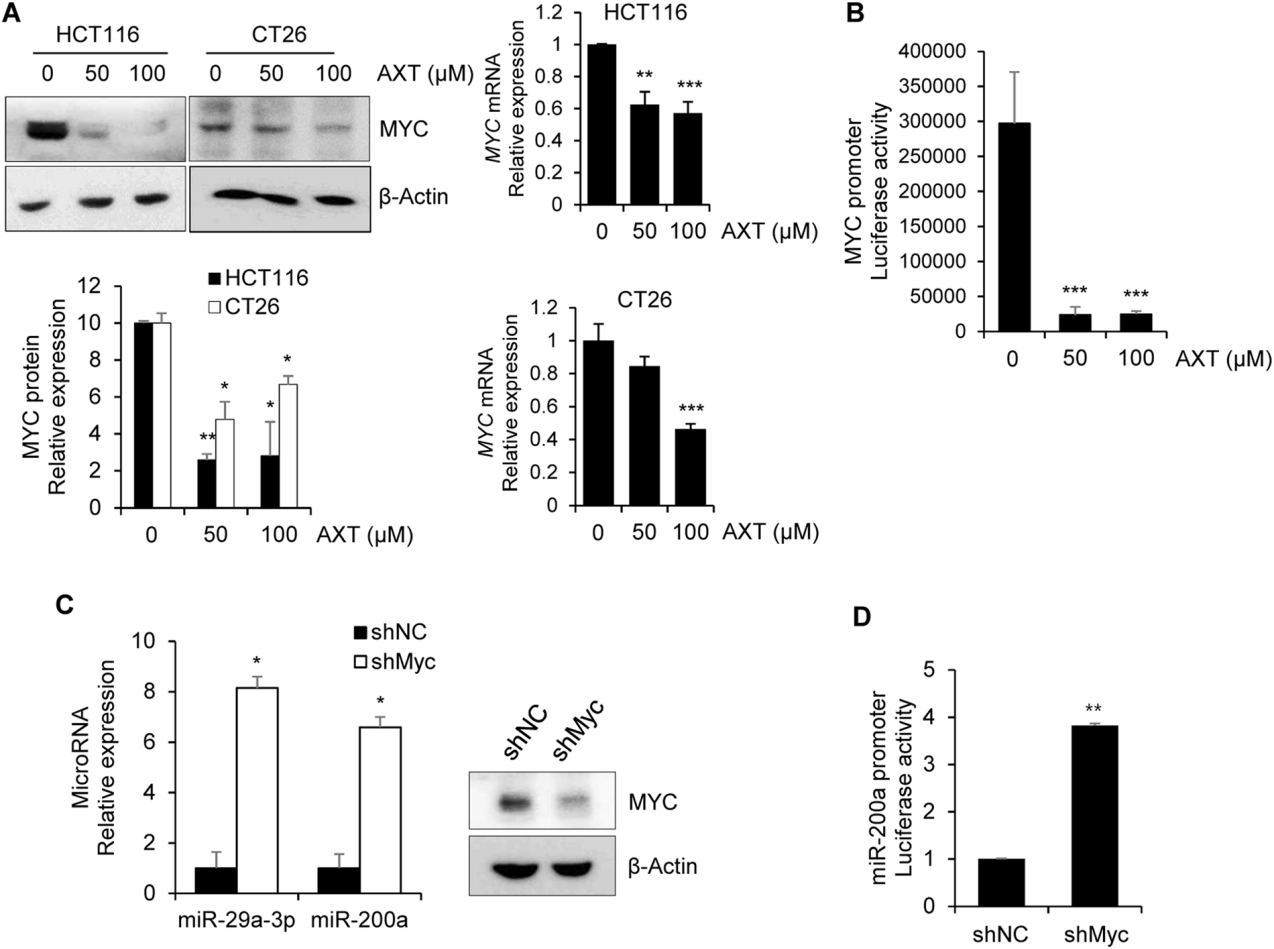
Astaxanthin negatively regulates MYC transcription factor at the transcriptional level. (**A**) To determine the expression level of MYC in AXT-treated colon cancer cells, protein and total RNA were purified, and examined with qRT-PCR and western blot. The band intensity was checked with Image J program, and normalized with β-actin. (**B**) To check the effect of AXT on the transcriptional regulation of *Myc*, CT26 cell was transfected with MYC promoter reporter plasmid, and treated with AXT. Relative luciferase activity was evaluated with luminometer. The β-galactosidase activity was measured to check the transfection efficiency. (**C**) To measure the expression levels of miR-29a-3p and miR-200a in *Myc* knockdowned HCT116 cells, the miRNAs were detected with qRT-PCR. Level of 18S RNA was measured for normalization. Knockdown of MYC was confirmed by western blot. (**D**) To confirm the effect of MYC on expression of miR-200a, miR-200a promoter luciferase construct was transfected into *Myc* knockdowned HCT116 cell. The relative luciferase activity was compared with control cells by luminometer. The β-galactosidase activity was measured to normalize the transfection efficiency. Results are generated as the mean ± SD from at least three replicated experiments. *P < 0.05; **P < 0.01; ***P < 0.001.

Previous studies reported that MYC oncogenic transcription factor represses the miR-29 and miR-200 family expression at a transcriptional level by direct interacting with canonical E-box MYC binding sites in promoter region, by which induces cancer metastasis^27–29^. Based on these studies, we examined whether AXT has inhibitory effect on the expression of MYC transcription factor. As shown in Fig. 4A, MYC protein and mRNA expression level were significantly decreased in AXT-treated colon cancer cells. To verify how AXT regulates MYC expression, we examined *Myc* promoter activity in AXT-treated CT26 cell. *Myc* luciferase activity was dramatically suppressed by AXT treatment, suggesting that AXT negatively regulates *Myc* expression at the transcriptional level (Fig. 4B).

Next, we verified the negative correlation between MYC and miR-29a-3p/miR-200a expression. The miR-29a-3p and miR-200a expression was measured in *Myc* knockdowned HCT116 cell by qRT-PCR (Fig. 4C). The expression of anti-metastatic miRs (miR-29a-3p and miR-200a) was recovered in *Myc* knockdowned cell. The knockdown efficacy of Myc was confirmed by western blot. More specifically, knockdown of *Myc* increases the miR-200a expression at the transcriptional level (Fig. 4D). Overall, these results suggest that AXT inhibits Myc expression at the transcription level, thereby restoring miR-29a-3p and miR-200a expression, and suppresses the metastatic ability of colon cancer cells.

### Astaxanthin suppresses the metastatic activity of colon cancer cell in *in vivo* model

**Figure 5:**
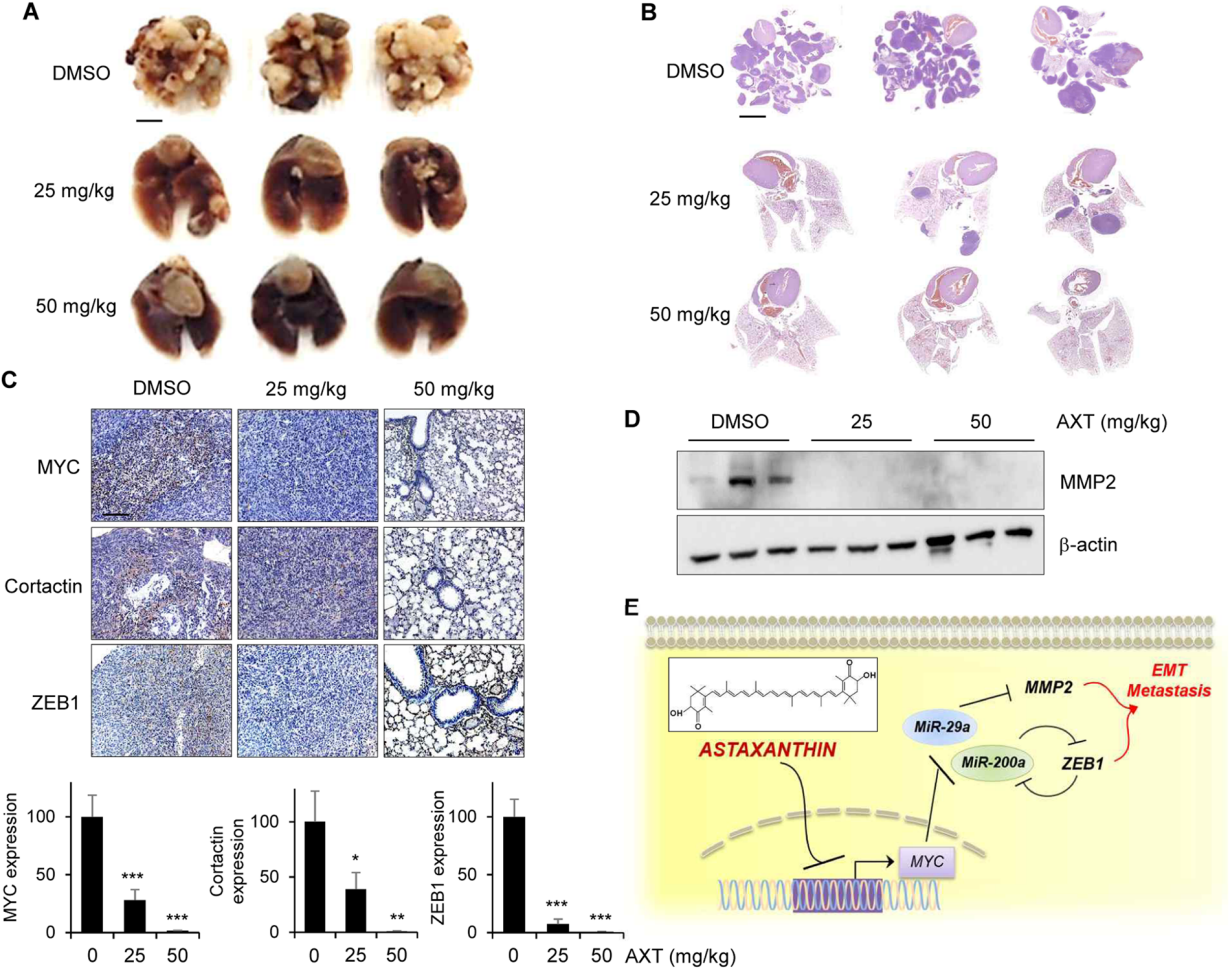
Astaxanthin suppresses the metastatic potential of colon cancer cell in *in vivo* model. (**A**) Representative images of lung metastasis after tail vein injection of CT26 cell (1 × 10^6^) into 6-week-old female nude mice (three mice in each group), and daily intraperitoneal injection of AXT of (25 or 50) mg/kg body weight for 4 weeks. (**B**) To check the lung metastasis of CT26 cells, H&E staining was performed with tissue samples that were treated with AXT. (**C**) Representative immunohistochemistry staining of MYC, Cortactin, and ZEB1 in lung tissues. Scale bar, 100 μm. (**D**) To determine the expression level of MMP2 in lung tissues, protein was isolated, and examined with western blot. The β-actin was used as loading control. (**E**) Proposal for suppressive activity of AXT in the metastasis of CRC. AXT increases the expression of anti-metastasis microRNAs (miR-29a-3p and miR-200a), thereby suppressing the expression of MMP2 and ZEB1, respectively. As a result, AXT suppresses the invadopodia formation and EMT process of CRC cells. Mechanistically, AXT shows anti-metastatic activity through the transcriptional repression of MYC transcription factor.

To determine whether AXT suppresses *in vivo* tumor metastasis, we injected CT26 cell (1 × 10^6^) through the tail vein. The mice were randomly seperated into three groups and treated with AXT (25 or 50 mg/kg) every day. The non-treated group developed lung metastasis rapidly in nude mice, whereas the metastatic growth of CT26 in lungs was completely suppressed in AXT-treated groups (Fig. 5A). Such difference was confirmed with whole-lung visualization by hematoxylin and eosin (H&E) staining of lung sections (Fig. 5B). Immunohistochemical analysis of MYC, Cortactin, and ZEB1 also showed AXT suppresses metastasis of colon cancer cells into lung (Fig. 5C). Finally, we checked the expression level of MMP2 in tumor tissues by western blot analysis. The expression of MMP2 was highly expressed in the non-treated group, but was decreased in the AXT-treated groups (Fig. 5D). Taken together, our results revealed that AXT suppresses the metastasis of colon cancer cell through the inhibition of invadopodia formation and EMT process.

## Discussion

Although many studies show that AXT is an interesting anti-cancer therapeutics through the modulation of multiple hallmarks of cancer, including proliferation, apoptosis, necrosis, autophagy, oxidation, and invasion, a little information is available on the function of AXT in colon cancer^16,30,31^. It has been reported that AXT suppresses cancer development targeting the JAK/STAT3 pathway and its downstream target gene expression of MMPs in a hamster model of oral cancer^30^. These MMPs (MMP2 and MMP9) are important components for degradation of the ECM during cancer metastasis. AXT was also found to suppress invasion in colon cancer by inhibiting the expression of MMPs, NF-κB, and Erk^16^. Furthermore, AXT reduces the activity of MMPs to suppress the metastasis of melanoma cancer cell in *in vitro* and *in vivo* model^32^. Although we presented the anti-metastatic activity of AXT with single treatment, it will be interesting to check the efficiency for combinatorial treatment with a wide range of chemotherapeutics to achieve better outcomes.

In this study, we report the anti-metastatic activity of AXT based on the suppression of invadopodia formation, EMT, consequent actin polymerization, matrix degradation, and the *in vivo* metastatic capacity of colon cancer cells. More specifically, we demonstrated that AXT restored the expression of miR-29a-3p and miR-200a by the inhibition of MYC oncogenic transcription factor. Through double-negative regulation, increased miRNAs suppress the expression of MMP2 and ZEB1 by direct targeting (Fig. 5E). We have identified several microRNAs (miRNAs) to elucidate the molecular link how AXT inhibits cancer cell metastasis by repressing MMP2 and ZEB1 expression. Previous report indicated that miR-29 family suppresses cancer angiogenesis, invasion, and metastasis in various tumors, through directly targeting the MMP2^21^. Bracken *et al*.^25^ reported that ZEB1 is an important and strong regulator of EMT and tumor metastasis, by which a double-negative feedback loop is established between ZEB1/2 and miR-200 family. On the basis of these results, we validated several putative miRNAs to find the target miRNAs that AXT regulates the expression of. Intriguingly, miR-29a-3p and miR-200a expression was increased in AXT-treated colon cancer cells in a dose-dependent manner.

The oncogenic *Myc* gene is upregulated in various human cancers, and can promote or suppress the transcription of carcinogenesis-associated genes. MYC also showed a marked effect on miRNA expression, which is widely downregulated, except with a miR-17~92 cluster, which is upregulated^33^. Much evidence demonstrated that several miRNAs are downregulated by MYC suggesting that miRNAs act as tumor suppressor^34,35^. For example, RAS targeting let-7 miRNA shows low expression in lung cancer^36^. The miR-34a having anti-carcinogenic activity is also suppressed by MYC^37^. Furthermore, MYC downregulates the miR-29 family to enhance the expression of AKT2 and CCND2, which is involved in AML development^38^. Previous reports demonstrated that MYC negatively regulated the miR-29 and miR-200 family through direct interaction on canonical E-boxes MYC binding sites in miR-29 (5′ CACGTG located at −261 and 5′ CACATG; −1317 from the transcription start site) and miR-200 (−110/+19) promoter region which resulted in elevated EMT^27–29^. Based on the above results, we confirmed whether MYC transcription factor suppresses miR-29a-3p and miR-200a expression. As a result, the expression of miR-29a-3p and miR-200a expression was increased in *Myc* knockdowned colon cancer cell, and AXT suppresses *Myc* expression at the transcriptional level. These results suggest that the increase in expression of miR-29a-3p and miR-200a by AXT occurs through suppression of the MYC transcription factor. Although many studies reported that AXT suppresses the expression of NF-κB and STAT3, transcription factors of MYC, to abrogate carcinogenesis in various cancer cells, the detail regulatory mechanisms are still elucidated^16,30,39^. Previous study has shown that master regulator of EMT process, Twist1 induces invadopodia formation through activating PDGFRα expression. The authors also mentioned that activation of Twist1 and invadopodia formation are required to degrade the basement membrane *in vivo*^9^. Like Twist1, MYC also positively regulated the EMT process, and was suppressed by AXT (Fig. 3). Although we did not check the effect of AXT on Twist1, AXT may function as anti-metastasis inhibitor through multiple mechanisms. Because AXT is a multi-functional carotinoid that is involved in various biological processes, many unidentified targets can be tested. To confirm the value of AXT as anti-metastasis drug, further studies are required to elucidate whether AXT modulates other specific target genes in the process of metastasis.

Invadopodia are actin-polymerized cell membrane adhesive structures to escape the basement membrane formed by metastasizing cancer cells. Therefore, ECM destruction is a critical process to initiating cancer invasion and metastasis^40,41^. Based on previous observations regarding the ability of invadopodia to extravasate the cancer cells through endothelial layer, we hypothesized that invadopodia inhibition could prevent the extravasation of cancer cells and subsequent tumor development. When we performed high-throughput drug screening based on invadopodia staining, it successfully identified the potential metastasis inhibitor (Fig. S1 of the SI). Therefore, it would be a valuable approach to screen more chemical libraries or natural compounds, to select novel therapeutics.

## Methods

### Antibodies and reagents

Synthetic AXT was purchased from Sigma-Aldrich, and dissolved in dimethyl sulfoxide (DMSO) to yield a 100 mM stock solution, and stored at −20 °C for future use. The primary antibodies (anti-E-cadherin (24E10), anti-Vimentin (5741) and anti-MMP2 (D204T)) were obtained from Cell Signaling. Anti-MYC (9E10) and anti-Cortactin (H-191) were obtained from Santa Cruz. Anti-β-actin (A5441) antibody was a product of Sigma Aldrich. Rhodamine Phalloidin (Thermo Fisher) was used for F-actin staining.

### Cell Lines and Culture

The human colon cancer cell HCT116 was cultured in RPMI-1640 (WelGENE, Korea), supplemented with 10% FBS, 1% penicillin, and streptomycin. Mouse colon cancer cell CT26 (ATCC) was cultured in DMEM (WelGENE) with 10% FBS, 1% penicillin, and streptomycin. Colon cancer cell lines were incubated at 37 °C in a 5% CO_2_ incubator.

### High-throughput screening for metastasis inhibitor

Colon cancer cells were plated onto 96-well chamber slides with 5,000 cells/well (Nunc™ Lab-Tek™ II Chamber Slide™). After 24 h, LOPAC1280 library (Sigma Aldrich) compound was treated with final concentration of 10 µM for a further 16 h. Cells were treated with Src family kinase inhibitor (SU6656) as positive control, or with vehicle (0.5% DMSO) as negative control. To verify the invadopodia structure, cells were fixed with fixation solution (3.7% PFA) for 15 min. Wells were aspirated and washed with PBS, then incubated with 0.1% Triton X-100 for 15 min. Cells were incubated with 5% BSA for 30 min, and stained with F-actin-phalloidin (1:500, Invitrogen) for overnight at 4 °C and DAPI (1:10,000) for 1 min. Images of invadopodia structure were taken and compared with Image J software (NIH) from at least three fields.

### qRT–PCR

To measure the expression level of specific genes, RNA was isolated from colon cancer cells with TRIzol reagent (Invitrogen). Table S1 of the SI shows the specific primers. Reverse-transcribed RNA with SuperScript II (Invitrogen) was analyzed with qRT-PCR using Prism 7900HT sequence detection system (Applied Biosystem) and SYBR-green Premix Ex-Tag II (Takara). Raw data were analyzed with comparative Ct method using *Cyclophilin* gene as normalization control. Expression of miRNA was checked with TaqMan^®^ MicroRNA Reverse Transcription Kit (Applied Biosystems), following the manufacturer’s method. Level of 18S RNA was measured for the normalization of miRNAs. Data were analyzed in at least triplicate, and expressed as mean ± S.D.

### Cell viability assay

The colon cancer cell lines (HCT116 and CT26) were plated onto 96-well plates (2 × 10^4^ cells/ml) and incubated for 24 h. Then, media were replaced with 100 μl of media containing indicated concentrations of AXT of (50, 100) µM for another 24 h. Then, cell viability was measured using the CCK8 and microplate reader at a wavelength of 450 nm.

### Western blot analysis

Protein lysates were extracted with a solubilization buffer (25 mM HEPES (pH 7.5), 1% Triton X-100, 5 mM EDTA, 10% glycerol, 150 mM NaCl, and a protease inhibitor cocktail) for 30 min and debris were removed with high-speed centrifugation at 10,000 × g for 10 min at 4 °C. For detecting target protein, protein samples were separated by SDS–polyacrylamide gel electrophoresis. Then, protein were detected with specific antibodies and visualized with chemiluminescence reagents according to the manufacturer’s method (Pierce).

### Generation of overexpression or knockdown colon cancer cell lines

To make the miR-29a-3p and miR-200a expression construct, the pCDH-CMV-MCS-EF1-copGFP (System Biosciences) system was used for cloning of miR-29a-3p and miR-200a DNA. Table S2 of the SI shows the sequences of each microRNA. After transfection into packaging cells, secreted viruses were isolated from the culture media using 0.45 µm filter, and infected the target cells with polybrene (8 µg/ml, Sigma-Aldrich). Expression of miRNAs were confirmed with miRNA-specific qRT-PCR after three times-repeated infections.

To make the knockdown system, pLKO lentiviral vector (shNC or shMyc) was introduced into Lenti293 cells (5 × 10^6^) with packaging DNAs. Table S3 of the SI lists the short hairpin RNA (shRNA) sequences targeting endogenous human *Myc*.

### Luciferase reporter assay

The potential miR-29a-3p interaction site of MMP2 gene was inserted into the pMIR-REPORT™ vector. For mutant form, the miR-29a-3p binding site of MMP2 (**T**GG**T**GCT) was replaced with mutant sequences (**A**GG**A**GCT) via site-directed mutagenesis. For *Myc* promoter assay, *Myc* promoter region was cloned into pGL3b vector. CT26 cells were cotransfected with the pGL3b-Myc reporter plasmid along with β-galactosidase as a normalizing control. After 24 h transfection, AXT was treated for another 24 h. The miR-200a promoter luciferase reporter assay was performed in a similar manner by cotransfecting with pGL3b-miR-200a promoter luciferase reporter plasmid and β-galactosidase into *Myc*-knockdown HCT116 cell. Luciferase activity was checked in triplicate with assay system and normalized with β-galactosidase activity (Promega).

### Fluorescent gelatin degradation assay

The invadopodium activity was compared with a QCM™ gelatin degradation activity based on manufacturer’s method (Millipore, ECM670). Briefly, cells were incubated on poly-L-lysine-coated 8-well chamber slides, followed by treatment with AXT (50 or 100) µM for another 24 h. Fluorescent image was taken and compared with Image J software from at least three fields. To confirm the invadopodia formation, cells were treated with 3.7% PFA, permeabilized with 0.1% Triton X-100 and blocked with 5% BSA. Then, actin was labeled with anti-phalloidin antibody to identify the invadopodia structure.

### Wound healing and cell invasion assays

To measure cell migration, cells were plated, and maintained until confluence in 6-well plates. After scratching with a pipette tip, cells were washed with PBS, and cultured in complete medium. Cell migration was quantified at 24 h after wounding. For the invasion assay, cells (1 × 10^4^) were placed in the top chamber (Thermo Scientific, 8 μm inserts), which contained DMEM with 0.1% FBS. Complete medium containing 10% FBS was placed in the bottom chamber. After incubation for 24 h, cells were fixed with 4% PFA, and stained with 0.005% crystal violet (Sigma-Aldrich). Noninvasive cells were removed with a cotton swab. Invaded cells were photographed under phase-contrast microscopy (Axioimager Z1, Zeiss), and data were quantified from triplicate experiments.

### *In vivo* metastasis analysis

BALB/c nu/nu mice (6 weeks old, (16–18) g, Orient Bio, Korea) were housed based on Institutional Animal Care and Use Committee-approved protocols of Gachon University. All animal experiments were performed in according to relevant guidelines and regulations. Mouse colon cancer CT26 cells (1 × 10^6^) were injected through tail veins of nude mice. The animals were randomized into 3 groups, with 8 animals per group. The experimental groups received daily intraperitoneal injections of AXT of (25 or 50) mg/kg. After 4 weeks injection, lung tissues were collected to check the metastasized colon cancer cells and perform the further analysis.

### Statistical analysis

All statistics analyzed with GraphPad Prism 5 program. Data are represented into mean ± S.D of three independent results. Analysis between different groups were performed with a 2-tailed paired Student’s *t*-test. Data were marked as statistically significant if the *P* value was ≤ 0.05.

## Supplementary information

Supplementary Information
