# Supplementary material for: Astaxanthin suppresses the metastasis of colon cancer by inhibiting the MYC-mediated downregulation of microRNA-29a-3p and microRNA-200a
Source: Sci Rep. 2019 Jul 1;9:9457. doi: 10.1038/s41598-019-45924-3 (PMC6603017; doi:10.1038/s41598-019-45924-3)
Supplement: Supplementary file 1 — Supplementary Information [file 41598_2019_45924_MOESM1_ESM.docx]

**Supplementary Information**

**Astaxanthin suppresses the metastasis of colon cancer by inhibiting the MYC-mediated downregulation of microRNA-29a-3p and microRNA-200a**

Hye-Youn Kim^1^, Young-Mi Kim^2^ & Suntaek Hong^1,2^

^1^Laboratory of Cancer Cell Biology, Department of Biochemistry, School of Medicine, ^2^Department of Health Sciences and Technology, GAIHST, Gachon University, Gachon University, Incheon 21999, Republic of Korea

Correspondence and requests for materials should be addressed to S.H. (email: sthong@gachon.ac.kr)

**
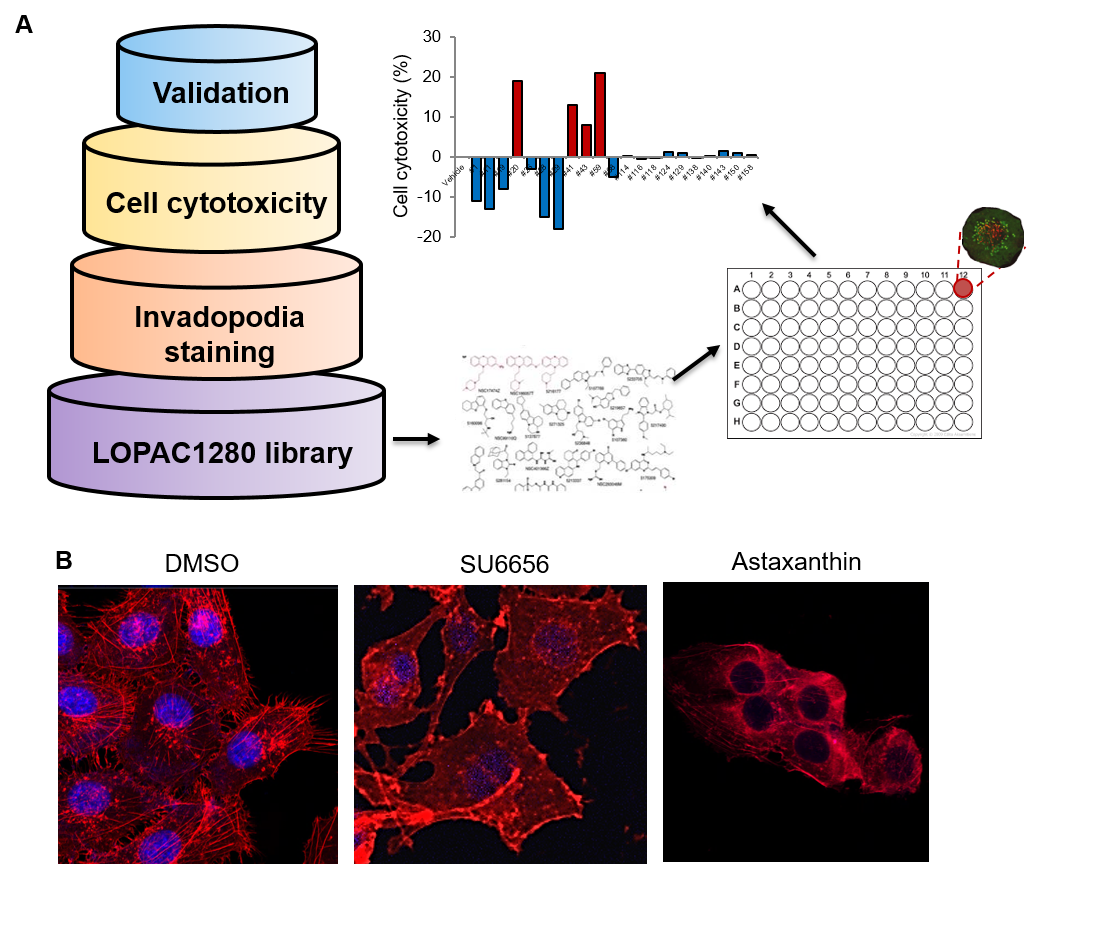
**

**Supplementary Figure S1. High-throughput chemical screening for the identification of invadopodia inhibitor**

(A) To establish the high-throughput drug screening, colon cancer cells were seeded into 96-well plate, and LOPAC1280 library was treated in duplicate, at a final compound concentration of 10 µM. Level of invadopodia formation was evaluated with F-actin staining by palloidin (red). Cytotoxic compounds were removed from the candidate compounds. To validate the effects of candidate compounds on invadopodia inhibition, wound assay, invasion assay, and gelatin-matrix degradation assay were performed. (B) Representative images for F-actin staining. Colon cancer cells were treated with DMSO and SU6656 as a negative and positive control, respectively. Scale bar, 50 μm.

**
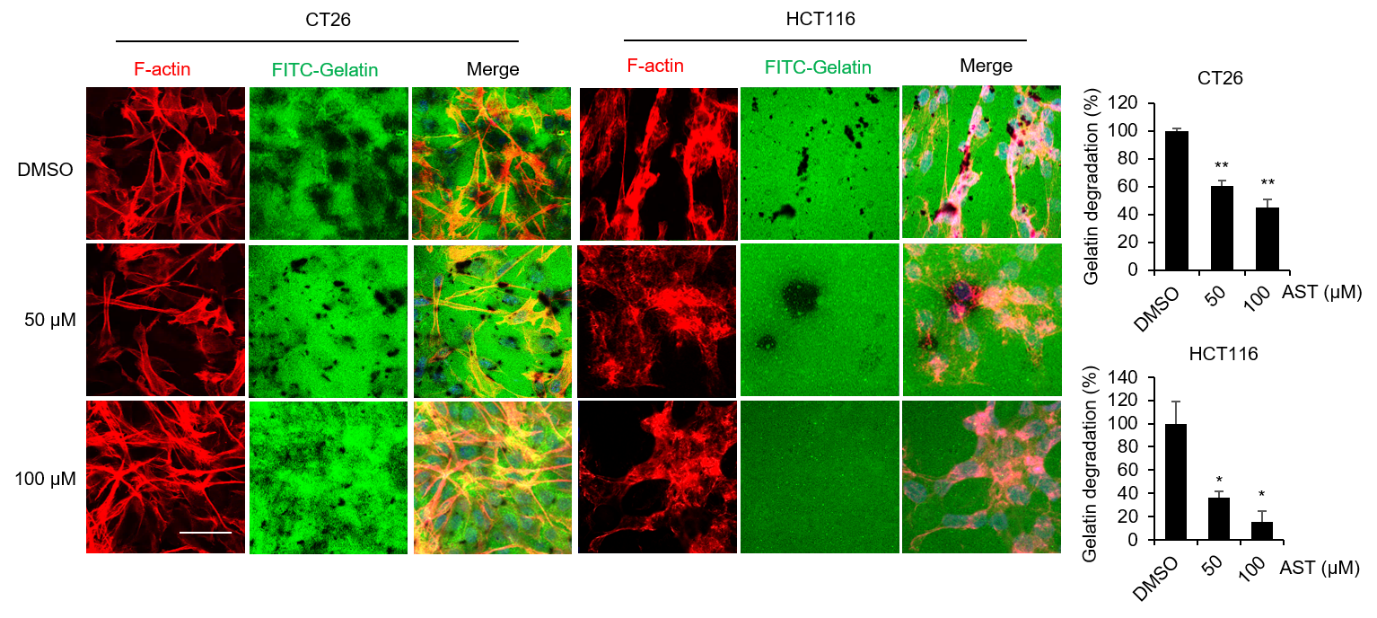
**

**Supplementary Figure S2. Suppression of invadopodia activity by Astaxanthin**

To confirm the invasive activity of colon cancer cells, slides were coated with FITC-conjugated gelatin (green). Colon cancer cells were cultured on the FITC-gelatin-coated coverslips for 48 h. To visualize F-actin, phalloidin (red) with DAPI (blue) were used to stain cells and nuclei, respectively. Punctuated black areas indicate degraded gelatin regions. Scale bar, 200 μm.


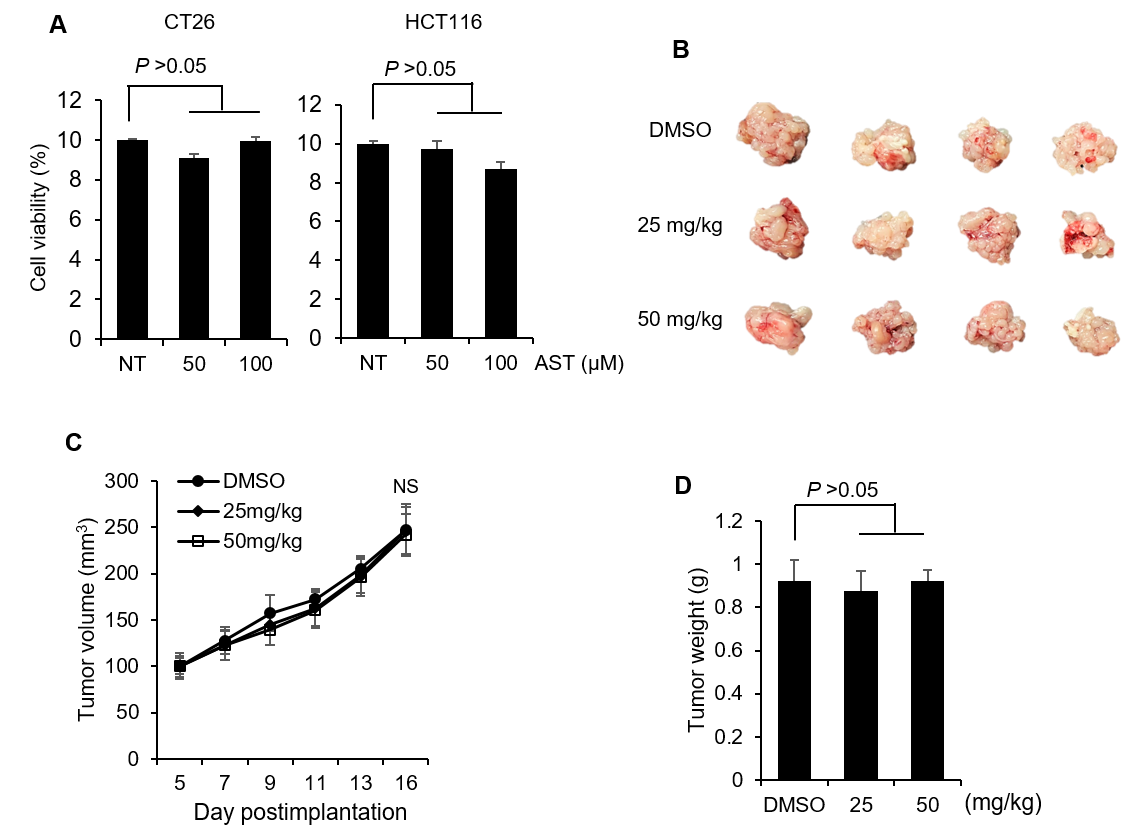


**Supplementary Figure S3. No growth inhibition by Astaxanthin**

(A) To measure the cell proliferation, MTT assay was performed in AXT-treated (50 and 100) µM colon cancer cells, compared with non-treated cells. (B) Representative images of tumors after subcutaneous injection of CT26 cell (1 × 10^6^) into 6-week-old female nude mice (8 mice in each group), and daily intraperitoneal injection of AXT of (25 or 50) mg/kg body weight for 16 days. (C) Mean tumor volume. (D) Mean tumor weight. Results are presented as mean ± SD from three independent experiments.

**
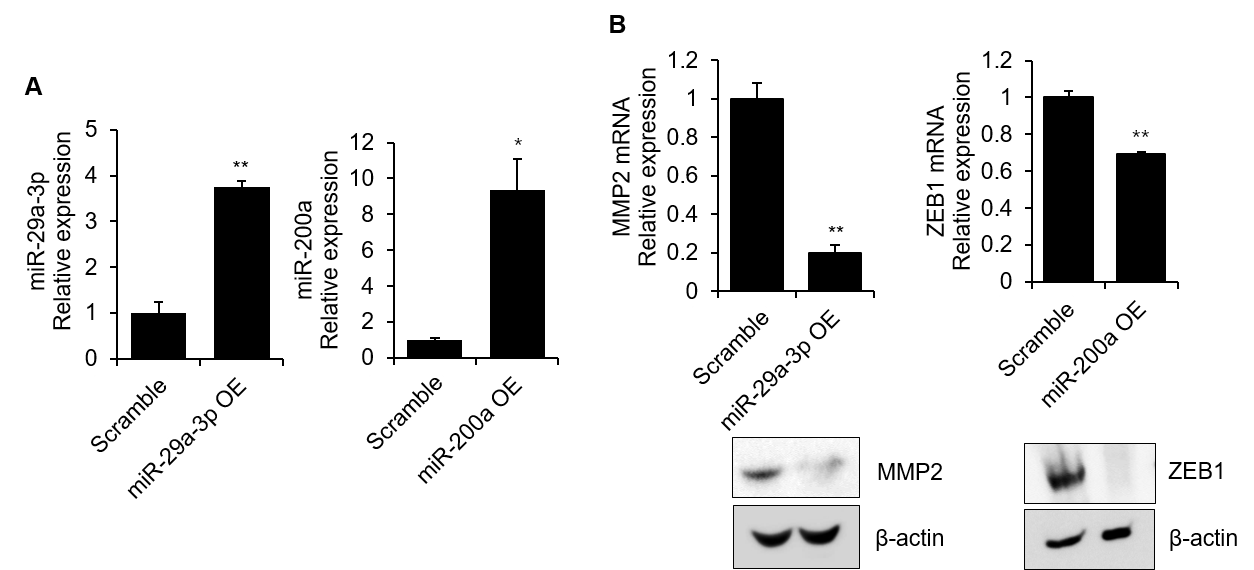
**

**Supplementary Figure S4. Generation of miR-29a-3p and miR-200a overexpressed cell lines**

(A) To generate miR-29a-3p and miR-200a overexpressed cells, lentiviral supernatant was infected into CT26 cell. Then, miR-29a-3p and miR-200a expressions were confirmed with miRNA-specific qRT-PCR, and normalized with 18S RNA. (B) qRT-PCR and western blot were performed to verify miR-29a-3p and mir-200a target genes, MMP2 and ZEB1, respectively. The β-actin was used as normalized control. Results are presented as mean ± SD from three independent experiments. *, *P* < 0.05; **, *P* < 0.01.


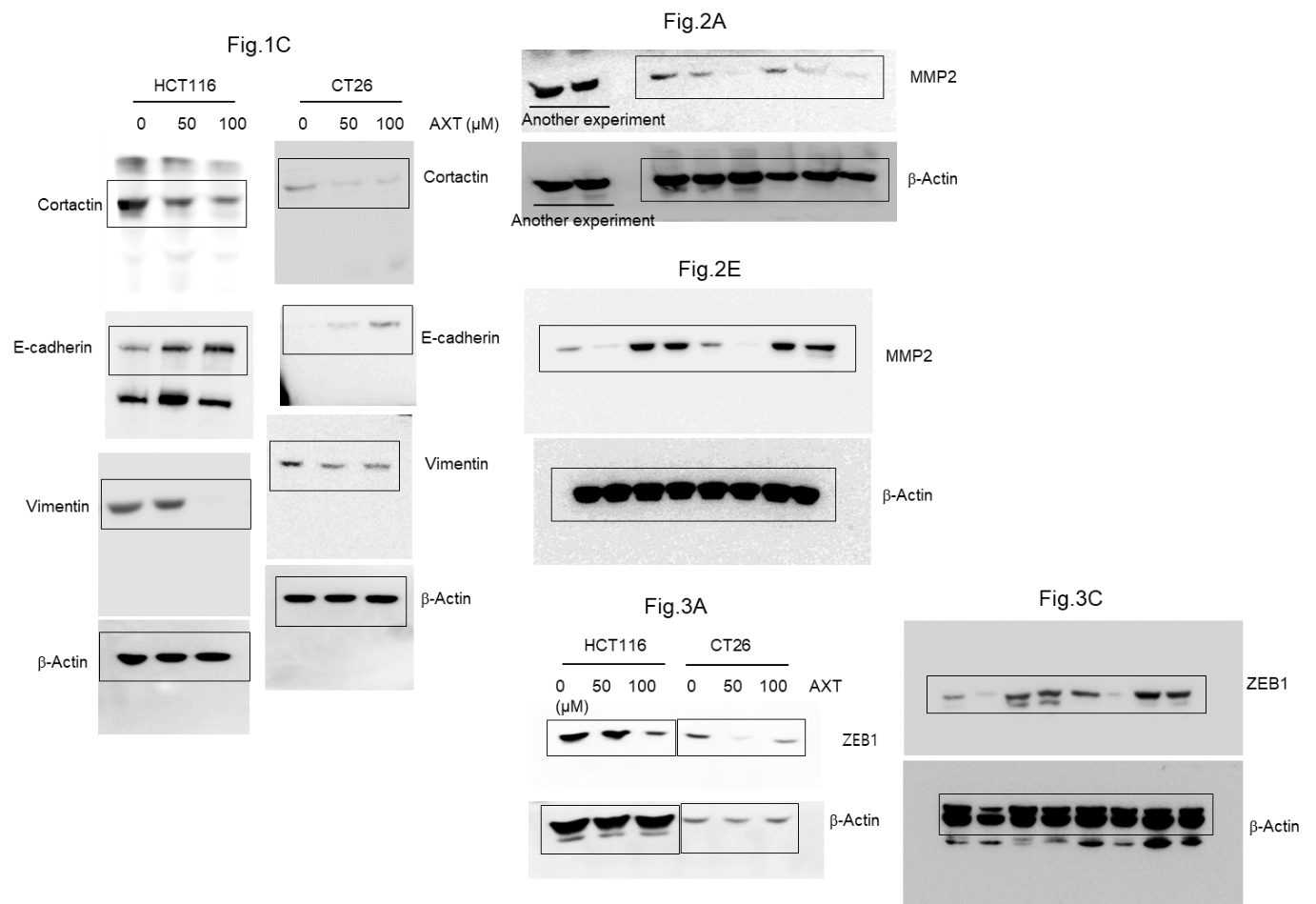


**
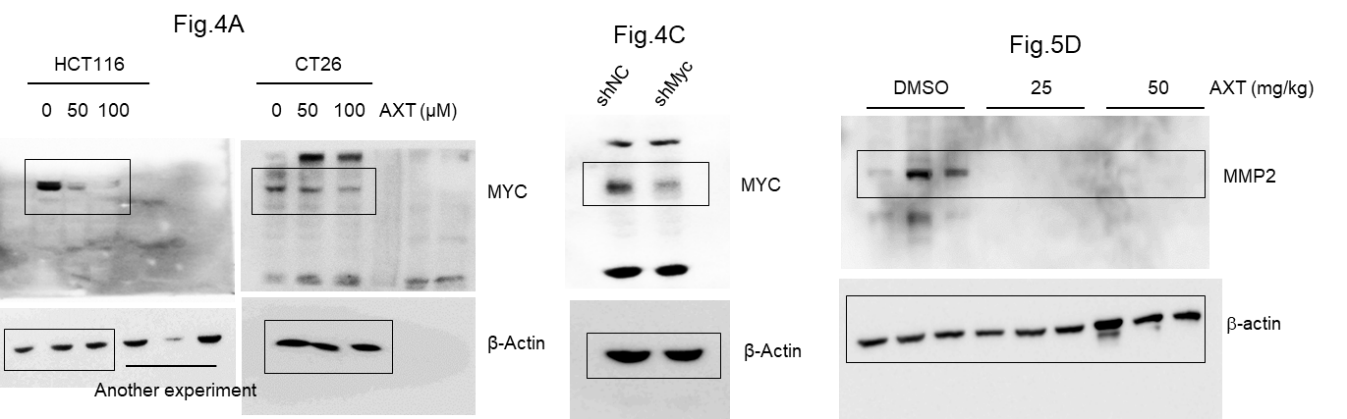
**

**Supplementary Figure S5. Full-length gels from Figures**

**
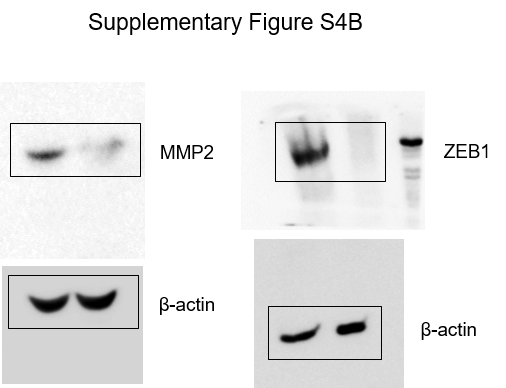
**

**Supplementary Figure S6. Full-length gels from Supplementary Figures**

**Supplementary Table S1. List of primer sequences for PCR**

| Gene | Forward (5´ to 3´) | | | Reverse (5´ to 3´) |
| --- | --- | --- | --- | --- |
| miR-29a-3p | GCGGCGG TAGCACCATCTGAAAT | | | ATCCAGTGCAGGGTCCGAGG |
| miR-200a | GCGGCGGC  ATCTTACCGGACAGT | | | ATCCAGTGCAGGGTCCGAGG |
| *hMMP2* | CCGTCGCCCATCATCAA | | | AGGTATTGCACTGCCAACTCTTT |
| *mMMP2* | GCACCATCGCCCATCATC | | TGCCAACTCTTTGTCTGTTTTAGG | |
| *hZEB1* | AAGAATTCACA  GTGGAGAGAAGCCA | | | CGTTTCTTGCAGTTTGGGCATT |
| *mZEB1* | GACACTCAC  TGTGTGCGACTCATA | | | GCTGGGCCAA  CTCTTAACAGA |
| *hMYC* | GCCACGTCTCCACACATCAG | | | TCTTGGCAGCAGGATAGTCCTT |
| *mMYC* | GTCTTTCCCTACCCGCTCAAC | | | GTGGAATCGGACGAGGTACAG |
| *hCyclophilin* | | TGAGCGCAAGTACTCCGTGT | | TCCACATCTGCTGGAAGGTG |
| *mCyclophilin* | | TGCCGGAGTCGACAATGAT | | TGGAGAGCACCAAGACAGACA |
| 18S RNA | ACCGCAGCTAGGAATAATGG | | | GCCTCAGTTCCGAAAACCA |
|  | | | | |

**Supplementary Table S2. List of primer sequences for precursor-microRNA overexpression used in this study**

| **miRNA** | **Sequence** |
| --- | --- |
| miR-29a-3p-F | GATC GAA TTC TTG CTT TGC ATT TGT TTT CTT AGT T |
| miR-29a-3p-R | GATC GGA TCC ACT CAT TCC ATT GTG CCT GGG TTA A |
| miR-200a-F | GATC GAA TTC CTG TGT GCA GTC TCA GGG CCC CCA A |
| miR-200a-R | GATC GGA TCC GTC TGC TGG CCC CGC TCG GCC CTC C |

**Supplementary Table S3. The target sequence of shMYC used in this study**

| Gene | Target sequences (5´ to 3´) |
| --- | --- |
| human shMYC | TGGAGATGATGACCGAGTTAC |
